# MicroRNA 146a (miR-146a) Is Over-Expressed during Prion Disease and Modulates the Innate Immune Response and the Microglial Activation State

**DOI:** 10.1371/journal.pone.0030832

**Published:** 2012-02-17

**Authors:** Reuben Saba, Shantel Gushue, Rhiannon L. C. H. Huzarewich, Kathy Manguiat, Sarah Medina, Catherine Robertson, Stephanie A. Booth

**Affiliations:** 1 Molecular PathoBiology, National Microbiology Laboratory, Public Health Agency of Canada, Winnipeg, Manitoba, Canada; 2 Department of Medical Microbiology and Infectious Diseases, University of Manitoba, Winnipeg, Manitoba, Canada; The Scripps Research Institute Scripps Florida, United States of America

## Abstract

Increasing evidence supports the involvement of microRNAs (miRNAs) in inflammatory and immune processes in prion neuropathogenesis. MiRNAs are small, non-coding RNA molecules which are emerging as key regulators of numerous cellular processes. We established miR-146a over-expression in prion-infected mouse brain tissues concurrent with the onset of prion deposition and appearance of activated microglia. Expression profiling of a variety of central nervous system derived cell-lines revealed that miR-146a is preferentially expressed in cells of microglial lineage. Prominent up-regulation of miR-146a was evident in the microglial cell lines BV-2 following TLR2 or TLR4 activation and also EOC 13.31 via TLR2 that reached a maximum 24–48 hours post-stimulation, concomitant with the return to basal levels of transcription of induced cytokines. Gain- and loss-of-function studies with miR-146a revealed a substantial deregulation of inflammatory response pathways in response to TLR2 stimulation. Significant transcriptional alterations in response to miR-146a perturbation included downstream mediators of the pro-inflammatory transcription factor, nuclear factor-kappa B (NF-κB) and the JAK-STAT signaling pathway. Microarray analysis also predicts a role for miR-146a regulation of morphological changes in microglial activation states as well as phagocytic mediators of the oxidative burst such as CYBA and NOS3. Based on our results, we propose a role for miR-146a as a potent modulator of microglial function by regulating the activation state during prion induced neurodegeneration.

## Introduction

Prion diseases, or Transmissible Spongiform Encephalopathies (TSEs), belong to a group of progressive neurodegenerative conditions that affect the nervous system in humans and animals. They can have a genetic etiology as well as occurring sporadically without any known risk factors or gene mutations. Uniquely, they are able to be transmitted in some instances, through foods and medical products contaminated with infectious prions. Disease progression is associated with the conversion of a normal cellular protein (PrP^c^) into an abnormal isoform associated with pathogenesis (PrP^Sc^), which in turn can build up in the brain as deposits in a similar fashion to amyloid in Alzheimer's diseased brains [1]. As is the case in other neurodegenerative conditions, such as Alzheimer's disease, one of the pathological features of disease is the activation of the brain's resident immune cells, the microglia, and the accumulation of astrocytes. In many instances, this feature can be detected prior to clinical symptoms and other signs of neurodegeneration, such as spongiosis and neuronal cell death become apparent [2], [3].

Microglia are normally quiescent but when activated they may undergo morphological changes, proliferation, chemotaxis and produce numerous cytokines and chemokines involved in inflammatory and immunomodulatory responses [4]–[6]. Such an inflammatory milieu has been recognized in the prion infected brain for some time; however, whether the activation of this innate immune response is beneficial or harmful in prion-like pathologies is still unknown. Increasing evidence suggests that microglial cells may be multi-functional, playing roles in brain tissue repair and neurogenesis, as well as in immunity [7].

In prion disease, microglia appear to express low levels of pro-inflammatory cytokines during chronic neurodegeneration but may be in a “primed” state [8]. The specific stimuli and signaling pathways that lead to these modulations of functions, and the mechanisms by which microglial activation influences chronic neurodegeneration, are as yet unknown. The contribution of toll-like receptors (TLRs), a family of proteins that are central players in the stimulation of innate immune responses, has been investigated in relation to prion disease progression. Upon recognition of their ligands, TLRs transduce intracellular signals via intermediary proteins including MyD88, TIRAP, TRIF, and TRAM, and signaling molecules such as IRAK4, IRAK1, and TRAF6. These signals translate to the production of cytokines and prostaglandins, and to genes involved in the generation of reactive oxygen intermediates required for the phagocytic process.

Un-methylated CpG DNA, which is an agonist of TLR9 signaling, has been shown to prolong the scrapie incubation period in mice, suggesting that innate immune activation can interfere with prion disease progression [9], [10]. Subsequently, transgenic mice with defective TLR4 signaling causing them to be hyporesponsive to lipopolysaccharide (LPS) were found to exhibit a significantly accelerated rate of prion disease development in comparison to their wild-type counterparts [11]. Interestingly, the anti-prion mechanisms induced by TLR4 signaling appear to be functional in the CNS rather than the periphery, as the rate of disease acceleration is similar in mice infected by both intra-peritoneal and intra-cerebral routes. Pathogenic changes in prion disease appear to be independent of MyD88, a signaling intermediate for several TLR family members, as ablation of this molecule did not lead to changes in the incubation periods, and neuropathological profiles of scrapie-infected mice [12].

Recent evidence suggests that small RNA molecules (microRNAs) are capable of regulating gene expression post-transcriptionally and play important roles in modulating the response of immune cells to stimuli [13]. MicroRNAs (miRNAs) are evolutionary conserved, non-coding, highly abundant small RNAs approximately 22 nucleotides long [14]. They are able to silence mRNA expression by sequence specific binding within the 3′UTR of protein-coding mRNA which results in either cleavage of the message or translational repression [15]. One miRNA in particular, miR-146a, has been shown to play an important role in the modulation of the innate-immune response. MiR-146a can directly down-regulate the production of pro-inflammatory cytokines by acting as a negative-feedback effector of the inflammatory signaling pathway initiated by NF-κB [16]. Recent work in our own laboratory showed the amount of miR-146a in the brain of scrapie infected mice to be significantly increased in comparison with age-matched control mice [17]. MiR-146a functionality has been explored in macrophages but its role in neurodegeneration has not yet been completely elucidated. We hypothesized that given the immunomodulatory role ascribed to miR-146a in macrophages; up-regulation of miR-146a in scrapie infected brain may reflect a role in keeping the pro-inflammatory response of microglia to prion replication and degeneration “in-check”.

## Materials and Methods

### Ethics statement

All procedures involving live animals were approved by the Canadian Science Centre for Human and Animal Health - Animal Care Committee (CSCHAH-ACC). All protocols were designed to minimize animal discomfort. The approval ID for this study was #H-O8-009.

### Biological material

CD-1 mice were inoculated intracerebrally with 20 µl of 1% brain homogenate from mice infected with the RML strain of mouse adapted scrapie. At the onset of clinical symptoms, 6 mice per sample group were sacrificed and hippocampus or cerebellar tissue used for RNA extraction. An equal number of age-matched, mock-infected mice (inoculated with a 1% brain homogenate of healthy CD-1 mice) were used as controls for each group.

### Cell culture

Two murine microglial cell lines were employed in this study, BV-2 and EOC 13.31 cells. The BV-2 cells were kindly provided by Dr. Yoon-Seong Kim (Department of Neurology/Neuroscience, Weill Medical College of Cornel University, New York) and the EOC 13.31 cells were obtained from the American Type Culture Collection Centre (ATCC). Both cell lines were grown and maintained in DMEM (ATCC) supplemented with 10% heat-inactivated FBS (Sigma) in a humidified incubator containing 5% CO_2_ at 37°C. However, the EOC 13.31 cells were additionally supplemented with 20% LADMAC conditioned media. LADMAC conditioned media was made from LADMAC cells purchased from ATCC that secrete CSF-1 as per manufacturer's instructions.

### TLR stimulation and neutralization of TLR2

2×10^5^cells/well were plated onto 24-well plates and incubated overnight in a humidified incubator containing 5% CO_2_ at 37°C. Cells were then stimulated for various time points with various concentrations of two types of LPS (*E. coli* 055:B5, Sigma)(*E.coli*, serotype EH100 (Ra), Enzo Life Sciences) or mock-treated cells as negative controls. To check the purity of both LPS stocks and that signaling was indeed through TLR4, cells were incubated for 30 minutes with various concentrations of purified monoclonal antibody to mouse (mAb mTLR2) (Invitrogen) followed by 24 hours stimulation with 100 ng/ml LPS. TLR2 stimulation was performed following incubation of cells in a humidified incubator containing 5% CO_2_ at 37°C for 8 hours at which time they were stimulated with 10^8^ cells/ml of heat-killed Listeria monocytogenes (HKLM) (Invitrogen).

### Mir-146a over-expression/knock-down

Over-expression of miR-146a was mimicked using a lipid-based reverse transfection system (siPORT™ NeoFX™ (Ambion)) following manufacturer's instructions. Briefly, pre-miR™ (precursor molecule for our over-expression strategy) miRNA-146a molecule along with the respective pre-miR™ miRNA precursor negative control #1 (Ambion) were diluted in OPTI-MEM® I medium (Invitrogen) to achieve a final concentration of 30 nM and then added with an equal volume of diluted siPORT NeoFX to each well of a 6-well plate. BV2 cells suspended in DMEM supplemented with 10% heat-inactivated FBS were overlaid in each well for a final total cell count of 2×10^5^ cells/well. Cells were incubated in a humidified incubator containing 5% CO_2_ at 37°C for 8 hours at which time they were stimulated with 10^8^ HKLM cells/ml (Invitrogen) for 24 hours.

Knock-down of miR-146a was achieved using the same transfection agent as previously stated (Ambion). Briefly, anti-miR™ (anti-sense miRNA molecule) miRNA-146a molecule along with the respective anti-miR™ miRNA negative control (Ambion) were diluted in OPTI-MEM® I medium to achieve a final concentration of 50 nM. The remainder of the procedure was followed the same way as previously stated for the over-expression studies including stimulation with HKLM. Additional negative controls included mock-transfected and mock-stimulated cells.

### Cytokine quantitation

To determine the levels of interleukin-6 (IL-6) and Granulocyte Macrophage - Colony Stimulating Factor (GM-CSF) secreted from the cells, supernatant was collected and a solid phase ELISA (Quantikine Mouse IL-6 or GM-CSF (R&D Systems)) was performed according to manufacturer's instructions.

### RNA isolation and miRNA quantitative real time-PCR (qRT-PCR)

To analyze miRNA expression, total RNA was isolated from cell culture using the miRVana miRNA Isolation kit (Ambion) according to manufacturer's instructions. MiRNA expression was measured and quantified using the TaqMan® miR-146a miRNA Assays (Applied Biosystems) according to the manufacturer's protocol. MiRNA expression was normalized to the U6 snRNA level which was expressed at high levels with negligible variance under the experimental conditions used in our study. Fold change was determined using 2^−ΔΔCt^ method.

### MiRNA microarray

The miRNA microarray was fabricated in-house. Low molecular weight (LMW) RNA (<200 nucleotides in length) was extracted from the cells passaged 3–4 times. The LMW RNA was then divided into equal halves, with one half being labeled with AlexaFluor 555 and the other half with AlexaFluor 647, for self/self hybridization experiments on the miRNA microarray. Hybridizations for each cell line were performed in quadruplicate. The criterion that was used for the inclusion of a miRNA in the overall repertoire of a cell line was correlation in spot intensity between the dye spots for a particular miRNA and intensity values stronger than a minimum arbitrary value of 10.

### Generation of amplified (aRNA), labeling and purification

The production of aRNA from total RNA was obtained using the AminoAllyl MessageAmp^II^ kit (Ambion) protocol according to the manufacturer's instructions. In brief, one round of amplification from total RNA samples was performed for each treated and control sample in order to generate enough target material for a dual-colour competitive hybridization. 1000 ng of total RNA was used to make the first cDNA strand. Once aRNA synthesis was complete, it was purified and split into two samples (∼10 µg of aRNA per sample) for dye-swapping experiments before vacuum drying in order to account for dye bias in the experiment. Once each sample dried, they were resuspended in coupling buffer (Ambion) and one sample labeled with an Alexa-Fluor 555 carboxylic acid succinimidyl ester dye (Invitrogen, Molecular Probes) and the other with Alexa-Fluor 647 carboxylic acid succinimidyl ester dye (Invitrogen, Molecular Probes) each made up in DMSO. Once the aRNA was labeled, it was purified and quanitated using a nanodrop spectrophotometer (Agilent technologies).

### Microarray slide hybridization

Each labeled aRNA sample was prepared, fragmented, and hybridized to an Agilent whole mouse genome 4×44K (Agilent) commercially manufactured array according to Agilent technologies manufacturer's instructions for two-color microarray-based gene expression analysis (version 5.0.1 August 2006) for 4×44K arrays. The arrays were hybridized for a total of 17 hrs at 65°C with vertical rotation.

### Microarray slide washing and scanning

The slides were washed according to Agilent's manufacturer's protocol for 4×44K arrays and scanned with the Agilent Microarray Scanner system with surescan technology (v.6.3) using the scanner settings for the 4×44K array format according to Agilent technologies manufacturer's instructions.

### Data analysis

The .TIFF images were uploaded into Feature Extraction Software v9.1 (Agilent technologiesG2567AA), spots were identified, and the grids constructed using the GE2-v5_91_0806 protocol and the 014868_D_20060807 grid file for “whole mouse genome” 4×44K expression arrays. Background calculations using spatial and multiplicative detrending and normalization using linear and lowess were performed by the Feature Extraction software. The p-value for differential expression was 0.01 and the resulting gene list was provided as log10 ratios of red (AlexaFlour 647)/green (AlexaFlour 555). Statistical analysis was performed using statistical analysis of microarrays (SAM) [18]. All microarray data is MIAME compliant and the raw data has been deposited to the Gene Expression Omnibus (GEO) under accession numbers: [GSE17759].

## Results

### MiR-146a is consistently up-regulated in the brain's of prion infected mice

We recently reported that miR-146a is amongst a select group of miRNAs up-regulated in the whole brains of mice infected with scrapie [17]. Quantitative real time-PCR (qRT-PCR) analysis using TaqMan® miRNA probes, specific for mouse miR-146a, revealed that this increase in expression is consistently found in mice clinically infected with a number of scrapie strains (22A, RML, and BSE) in comparison to age matched, mock-infected mice (Table 1). Between a 3- and 16-fold increase in the level of expression was detected in RNA extracted from whole brain, hippocampus and cerebellar tissue samples.

**Table 1:** Expression levels of miR-146a in brain tissue of mice infected with mouse-adapted scrapie or BSE as determined by quantitative RT-PCR (TaqMan® qRT-PCR) (relative quantitation was determined using the 2^−ΔΔCt^ method).

Scrapie strain	Mouse strain	Tissue	Fold increase (infected vs. uninfected)	p-value (t-test) (infected vs. uninfected)
22A	VM	Whole brain	3.3	9.4×10^−2^
RML	CD1	Hippocampus	8.3	3.5×10^−2^
RML	CD1	Cerebellum	16.3	6.8×10^−3^
BSE	CD1	Cerebellum	10.0	7.6×10^−2^

**Figure 1:**
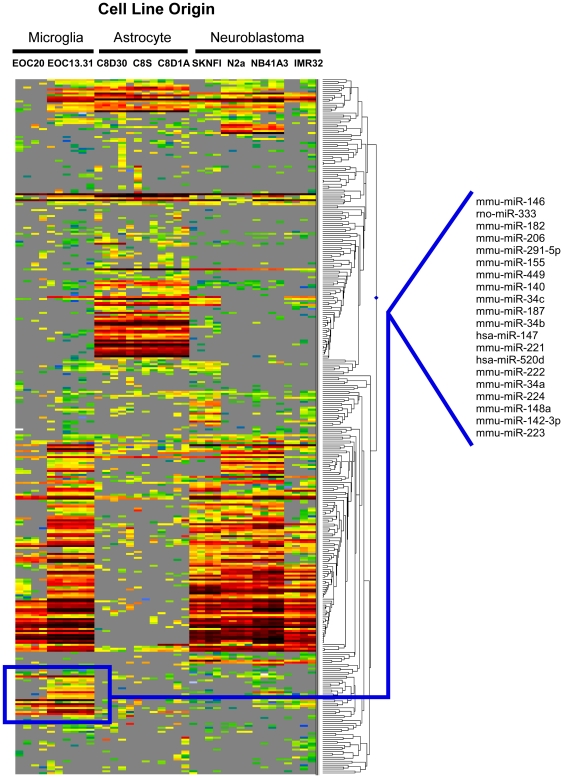
Hierarchical cluster plot generated from miRNA expression profiling of a repertoire of a variety of CNS cell lineages (neuronal, microglia, astrocytes). A microglia specific cluster of miRNAs is indicated. Red indicates high levels of miRNA expression, green low levels and gray indicates expression that was undetected by the microarray used.

MiR-146a functionality has been explored in macrophages but its role in neurodegeneration has not yet been elucidated [16]. PrP^Sc^ induces inflammatory responses by the brain's glial cells (astrocytes and microglia) by activating innate immunity pathways. Given the immunomodulatory role ascribed to miR-146a in macrophages, we hypothesized that expression in the brain would likely be enriched in astrocytes and/or microglia. *In situ* hybridization to detect miR-146a expression proved inconclusive in confirming this (data not shown). We therefore profiled RNA from multiple brain-derived cell lines of both mouse and human origin to conditionally determine the expression profiles of a number of miRNAs, including miR-146a. These cell lines included neuronal subtypes (N2A, NB41A3, SKNFI, IMR32), microglial subtypes (EOC 13.31, EOC 20) and astrocyte subtypes (C8D30, C8S, C8D1A). Cultured cells were passaged 3–4 times prior to the extraction of low molecular weight (LMW) RNA (<200 nucleotides in length), which was then labeled and hybridized to a miRNA microarray [19]. A representative hierarchical cluster plot to visualize this data is provided in Figure 1. Small clusters of neuronal-, astrocyte- and microglia-enriched miRNAs are also apparent. The neuronal cluster included miR-124, a miRNA that is especially abundant in neurons affirming the specificity of the methodology [20]–[22]. The microglial cell-line enriched cluster contained 20 miRNAs including miR-146a and a number of other miRNAs reportedly involved in inflammation, such as miR-155, miR-221 [23] and miR-147, a microRNA that is induced upon Toll-like receptor (TLR) stimulation and regulates murine macrophage inflammatory responses [24].

### The microglial surface receptor, TLR2, is significantly induced in the brain's of prion infected mice

MiR-146a is reported to be up-regulated in LPS stimulated human macrophages, however, the microglial surface receptor that is engaged leading to activation during prion pathogenesis is unknown [16]. We used a microarray analysis to determine which microglial surface receptors are over-expressed in the brains of an RML scrapie model of prion disease and may therefore play a pertinent role in activation. Brain tissue from the hippocampal and cerebellar regions of scrapie infected mice at clinical stages of disease consistently showed the significant up-regulation of the TLR2 gene (the most abundant TLR gene based on microarray probe intensity) as well as genes coding for the receptor accessory molecules LY96 and CD14 (Table 2). The only other TLRs we determined to be significantly up-regulated were TLR1 and TLR7. However, the mRNAs of other TLRs, including TLR4, were barely detectable in our study and so we could not determine whether there was an increase in expression of these receptors in the infected animals. Other receptors including Fc (FCGR1 and FCGR3) and complement receptors (ITGAM and ITGAX), as well as TREM2 a myeloid specific receptor triggered by amyloid to promote active phagocytosis, were also found to be over-expressed. Scavenger receptors (SCARB1, SCARB2 and AGER), except for CD68, did not show significant induction.

**Table 2:** Expression levels of microglial cell surface receptors in hippocampal tissue of RML infected, symptomatic mice as determined by microarray analysis.

Gene	Fold increase (infected vs. uninfected)	p-value (t-test) (infected vs. uninfected)
**Toll like receptors & co-receptors**		
TLR2	18.6	0.001
TLR7	5.8	0.034
TLR1	10.9	0.081
LY-96 (MD-2)	4.9	0.023
CD14	16.2	0.013
**Scavenger receptors**		
SCARB1	1.3	0.574
SCARB2	1.0	0.958
CD68	13.8	0.040
AGER	1.1	0.394
**Fc receptors**		
FCGR1	24.5	0.003
FCGR2	4.4	0.047
**Complement receptors**		
ITGAM	6.5	0.003
ITGAX	15.1	0.030
**Other microglial receptors**		
TREM2	13.2	0.018

### MiR-146a expression is induced in microglial cell lines by TLR2- and TLR4-agonists

**Figure 2:**
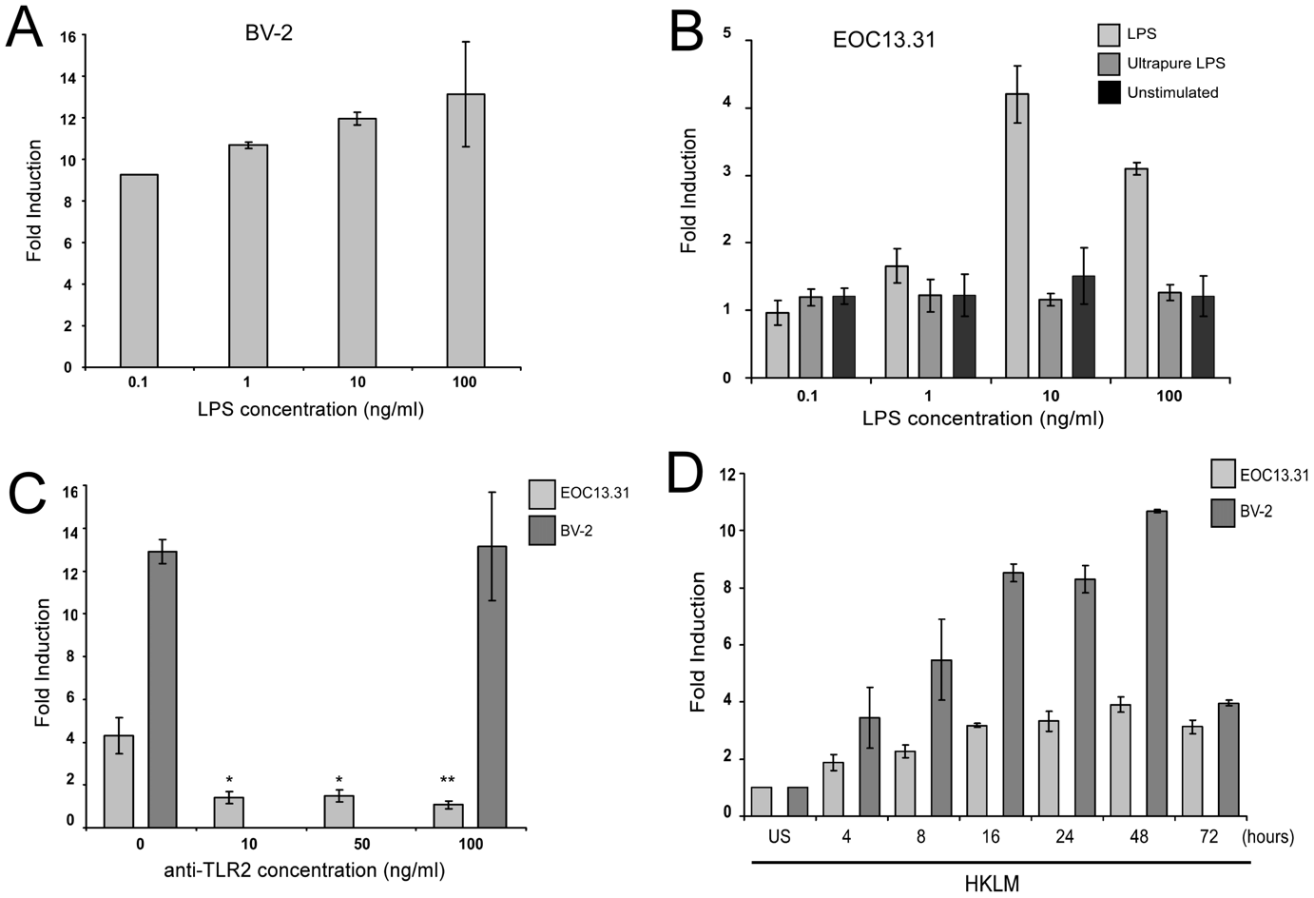
MiR-146a induction in microglia. **A.** LPS at concentrations ranging from 0.1–100 ng/ml was used to stimulate BV-2 cells. After 8 hours, RNA was collected and TaqMan® qRT-PCR was used to determine miR-146a expression relative to PBS treated (unstimulated) control cells. The experiment was performed in triplicate and the average fold induction is shown. **B.** EOC 13.31 cells were stimulated with 100 ng/ml semi-pure LPS, 100 ng ultra-pure LPS or PBS alone (unstimulated). RNA was collected after 8 hours and miR-146a expression was measured by TaqMan® qRT-PCR. Fold induction relative to untreated cells is shown. The experiment was performed in triplicate and the average fold change is shown. **C.** EOC 13.31 cells were incubated with increasing concentrations of an anti-TLR2 antibody for 30 minutes prior to stimulation with 100 ng/ml LPS. MiR-146a expression relative to mock-treated control cells was measured by TaqMan® qRT-PCR. Inhibition of miR-146a expression following anti-TLR2 antibody treatment was significant at all concentrations; 10 and 50 ng/ml * p<0.01, 100 ng/ml ** p<0.005. Treatment of BV-2 cells with 100 ng/ml anti-TLR-2 antibody prior to LPS stimulation failed to inhibit miR-146a induction. The experiment was performed in triplicate and the average fold change is shown. **D.** EOC 13.31 and BV-2 cells were stimulated with with 10^8^ heat-killed *Listeria monocytogenes* (HKLM) cells/ml. RNA was collected at various time-points over 72 hours and miR-146a expression measured by TaqMan® qRT-PCR. Fold induction relative to unstimulated cells (US) is shown. The experiment was performed in triplicate and the average fold change is shown.

Previous studies have shown that miR-146a is up-regulated in LPS stimulated human monocytic cell line THP-1, a cell line that expresses Fc and C3b receptors and is a common cell culture model for the study of macrophages [16]. We investigated whether a similar response was induced by LPS in a microglial-derived line, BV-2. This line is derived from murine microglial cells that were immortalized after infection with a v-raf/v-myc recombinant retrovirus. These cells share numerous properties with macrophages with respect to the antigen profile, their phagocytic capacity and antimicrobial activity [25]. Additionally, like microglial cells, they form spineous processes and express inwardly rectifying, but not outwardly rectifying K^+^ channels [25]. BV-2 cells were treated with LPS at varying concentrations (between 0.1–100 ng/mL) over a period of 8 hours. MiR-146a levels were determined by TaqMan® qRT-PCR and provided in Figure 2A. In general, the expression of miR-146a increased upon LPS stimulation to a maximum of 13-fold at 100 ng/ml. LPS signaling is mediated by TLR4 since mutations or deletions of the *Tlr4* locus completely abolish LPS-mediated signaling [26], [27]. To show that miR-146a induction is specifically mediated by the LPS/TLR4 signaling pathway we used a second microglial line, EOC 13.31 derived from C3H/Hej mice that has a spontaneous mutation in the TLR4 gene (*Tlr4^Lps-d^*) making it hyporesponsive to LPS [28]. We also observed significant up-regulation of miR-146a in EOC 13.31 cells which we hypothesized to be likely due to the presence of a contaminant in the particular LPS preparation in use, an observation previously reported by others [29]. Stimulation with an ultrapure LPS preparation did not evoke miR-146a up-regulation in EOC 13.31 cells confirming this was the most likely explanation (Figure 2B). Taganov *et al* (2006) previously showed that TLR2 stimulation by exposure to peptidoglycan and its synthetic analog, Pam3CSK4, produced an up-regulation of miR-146a in human monocytes in contrast to TLR3, TLR7 and TLR9 agonists. To test whether the crude LPS preparation in our laboratory contained agonists that could stimulate EOC 13.31 TLR2 receptors, we treated the cells with an anti-TLR2 antibody for 30 minutes prior to treatment with LPS. This treatment, in turn, prevented the induction of miR-146a in EOC 13.31 cells stimulated via the crude LPS preparation, but not similarly treated BV-2 cells (Figure 2C). Treatment with an ultra-pure TLR2 agonist, HKLM, resulted in an induction of miR-146a in both cell lines (Figure 2D). Given that TLR2 and TLR4 stimulation of microglial cell lines can lead to an increase in expression of miR-146a, potentially either one, or both, of these molecules are involved in the signaling that leads to the up-regulation of this miRNA in the brain's of prion infected mice. Nevertheless, the expression of TLR2 is much greater than TLR4 (below detection threshold) in the brain's of mice tested in this study.

### Temporal induction of miR-146a expression in microglia is kinetically distinct to that of TLR2 signaling-induced cytokines

**Figure 3:**
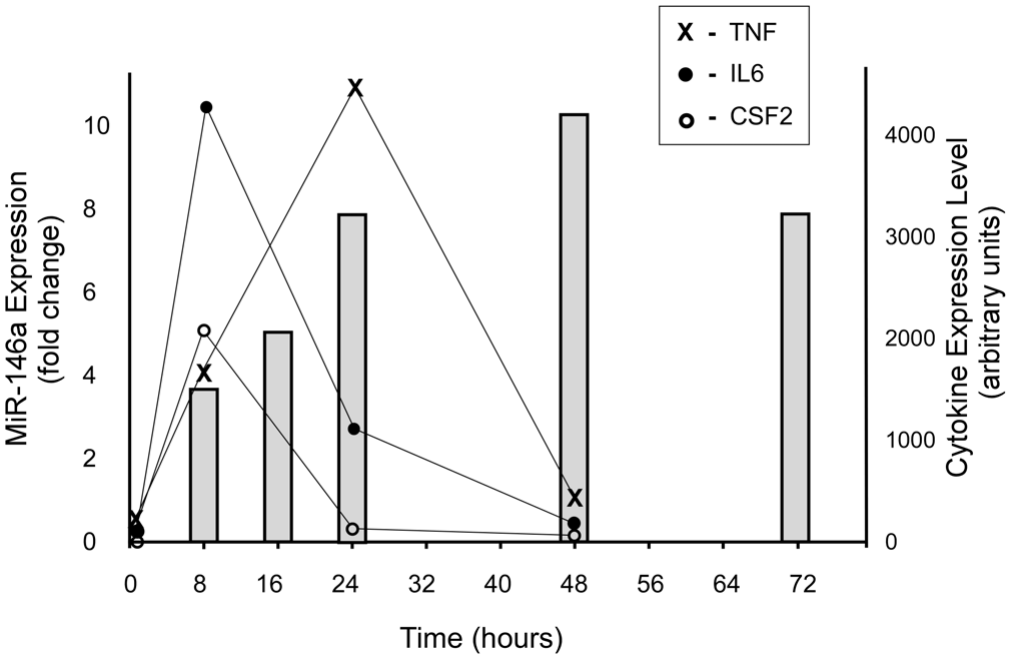
EOC 13.31 cells were stimulated with 100 ng/ml semi-pure LPS and temporal changes in cytokine gene expression relative to mock-treated control were measured by microarray analysis. MiR-146a expression relative to mock-treated control was measured by TaqMan® qRT-PCR and expression is shown as grey bars.

Agonists to TLR2 and TLR4 induce the rapid expression of cytokines which return to endogenous levels expeditiously. To determine whether the induction of miR-146a in BV-2 and EOC 13.31 cells followed a similar path we stimulated with an 100 ng/ml concentration of the semi-pure LPS preparation and measured expression relative to mock-treated control cells using TaqMan® qRT-PCR. Agonist treatment uniformly resulted in the significant induction of miR-146a expression that increased over time to a maximum at 48 hours post-stimulation, but was still elevated even at 72 hours post stimulation (Figure 3). This was is in contrast to the generally rapid induction of cytokine expression following LPS stimulation. We specifically followed the levels of transcripts coding for the cytokines, IL-6 and CSF2 (colony stimulating factor 2 or GM-CSF), and tumor necrosis factor (TNF). All three were found to peak well before 24 hours post-stimulation and had all returned to basal levels by 48 hours (Figure 3). These data suggest that induction of miR-146a may be secondary to the initial response triggered by the TLR agonist. This has been previously described in macrophages where miR-146a expression was found to be capable of dampening the immediate innate immune response mediated by TLR2 and TLR4 [16], [30], [31].

### Transcription of genes involved in protein synthesis, infection mechanism, immune cell trafficking, oxidative phosphorylation and cellular movement are modulated by miR-146a expression in microglia

**Figure 4:**
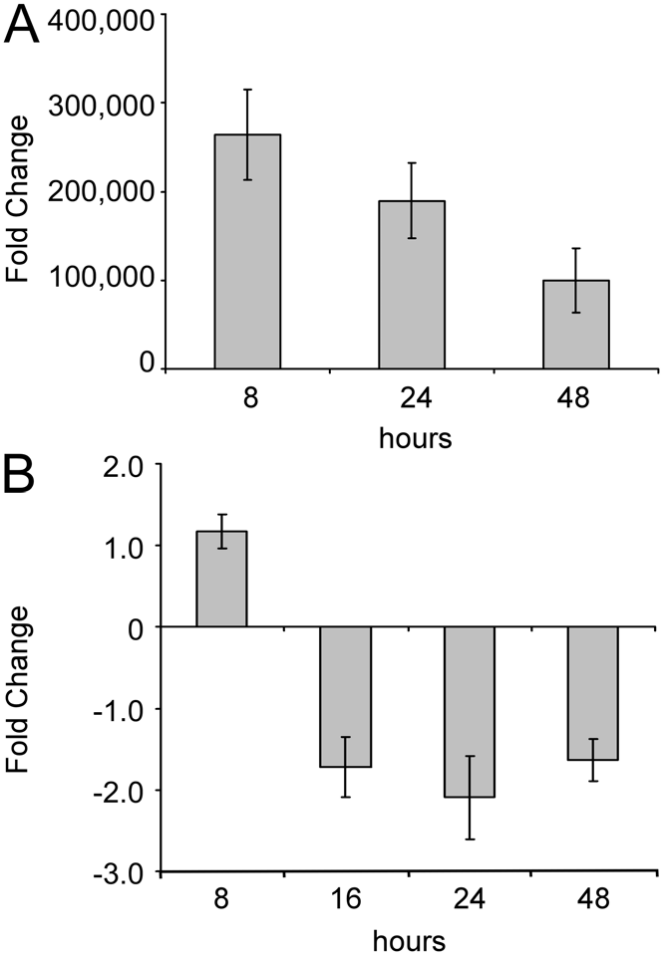
Altering the expression of miR-146a in microglia. **A.** MiR-146a over-expression as measured by TaqMan® qRT-PCR in microglia cells after 8, 24, and 48 hrs of treatment with 30 nM (previously optimized, data not shown) of transfected pre-miR-146a in comparison to 30 nM of transfected scrambled negative control miRNA. An average fold change and standard deviation were measured from triplicate experiments. **B.** MiR-146a knock-down as measured by TaqMan® qRT-PCR in microglia cells after 8, 24, and 48 hrs of treatment with 50 nM (previously optimized, data not shown) of transfected anti-miR-146a in comparison to 50 nM of transfected scrambled negative control miRNA. An average fold change and standard deviation were measured from triplicate experiments.

With the aim of identifying those biological functions under miR-146a control, we used a functional genomic strategy to determine the extent of transcriptional changes modulated by miR-146a expression in the microglial cell line EOC 13.31. This line was chosen rather than BV-2 due to high levels of transfected anti-miR (complementary to miRNA) and pre-miR (precursor miRNA or miRNA mimics) being detectable in the cells for much longer, up to 36 hours post infection, despite the fact that the transfection rates for both cells were similar (data not shown). This was likely due to differences in the proliferation rates exhibited by the two cell lines. While the BV-2 cell line divided very rapidly, at least once during a 24 hour period, EOC 13.31 cells doubled only every 48 hours. This enabled us to obtain maximal knock-down and over-expression of miR-146a, whilst allowing the cells to recover overnight following transfection prior to RNA extraction, therefore minimizing expression changes that might be due to the transfection procedure itself. Over-expression was achieved by transfecting 30 nM of miR-146a mimic (Figure 4A). We experimentally determined that knock-down of miR-146a in EOC 13.31 cells was optimal between 16 and 48 hours post-transfection upon transfection of 50 nM of miR-146a anti-miR (Figure 4B).

Over-expression of a miR-146a mimic for 24 hours resulted in significant deregulation of hundreds of genes; 1668 genes had ≥1.5-fold alteration of expression (FDR ≤2%) in comparison to EOC 13.31 cells transfected with a scrambled control RNA. Analysis of these data based on enrichment for biological functions was performed using the Ingenuity Pathway Analysis (IPA) tool, which revealed a significant number of the deregulated genes to be involved in cell morphological changes, cell signaling, RNA post-transcriptional modification and cell cycle regulation. Changes include the down-regulation of molecules such as ITGA4, ITGAV, S1004A, CCL17, CDKN1B, VEGFA and ERBB2 involved in cell movement, adhesion and shape change. Additionally, a broad spectrum of genes involved in antimicrobial activity were mostly down-regulated including some genes involved in signaling pathways from TLR and Fcγ receptor-mediated phagocytosis including HCK, LYN, PKCG1, SYK and ARF6. Complement factor H (CFH) and mannose-binding lectin serine peptidase 2http://www.genenames.org/data/hgnc_data.php?hgnc_id=6902 (MASP2), two proteins secreted into the bloodstream that play roles in the regulation of complement activation, were also deregulated. CFH and MASP2 are both involved in antibody-independent phagocytosis and are essential regulators of complement activation that restrict this innate defense mechanism to microbial infections. CFH has also been reported to be a direct target of miR-146a and potentially important in inflammation in Alzheimer's disease [32], [33]. Another significant group of genes that exhibited down-regulated expression incorporate those related to the oxidative burst phenomenon that is at play during phagocytosis. These included the genes NOS1, NOS2 and NOS3, nitric oxide synthases that are inducible by TLR stimulation and NOX4, a gene involved in H_2_O_2_ production. Significantly, the genes CYBA and CYBB proposed to be primary components of the microbicidal oxidase system of phagocytes, were also down-regulated, as was SOD3. SOD1, however, was up-regulated in our work. These isozymes are soluble cytoplasmic proteins responsible for the conversion of superoxide radicals to molecular oxygen and hydrogen peroxide.

A smaller group of up-regulated genes were identified following miR-146a over-expression. Ontological analysis determined the most significant group of 35 genes (p = 2.4×10^−9^) to be involved in RNA post-transcriptional modification. These genes included TNRC6A and TNRC6B, whose gene products are members of the trinucleotide repeat containing 6 protein family and function in post-transcriptional gene silencing through the RNA interference (RNAi) and miRNA pathways. They associate with messenger RNAs and Argonaute proteins in P-bodies.

Substantially fewer genes were dysregulated following transfection of miR-146a anti-miR and these were for the most part up-regulated. Ontological analysis revealed a significant number of these genes to be involved in protein synthesis, infection mechanisms, immune cell trafficking, oxidative phosphorylation and cellular movement. Ribosomal proteins were strikingly up-regulated resulting in a significance score in IPA of p = 1.13×10^−54^, similarly numerous mitochondrial cytochrome C oxidase subunits involved in oxidative phosphorylation were also up-regulated. Another interesting up-regulated gene was the ferritin light chain (FTL) which is involved in the protection of cells from oxidative stress. The gene DC-SIGN (CD209) was one of those innate immune related genes that exhibited increased expression following knock-down of miR-146a. DC-SIGN is a transmembrane receptor that recognizes numerous evolutionarily divergent pathogens ranging from parasites to viruses with a large impact on public health. Interestingly, knock-down of DICER1, required for miRNA processing has recently been shown to alter the levels of DC-SIGN in dendritic cells revealing potential regulation by miRNAs. TANK, a TRAF family member associated with NF-κB activation, was also up-regulated by miR-146a knock-down. TANK is activated by TRAF6, and appears to be part of a NF-κB regulatory loop. Previous studies have shown that TANK can act both in both positive- and negative-regulation of NF-κB and interferon pathways by positively regulating IRF3.

**Figure 5:**
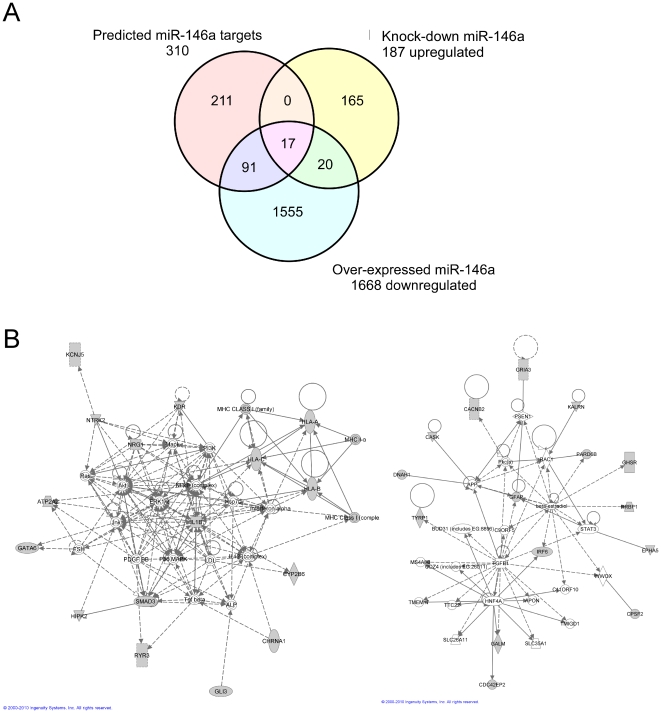
Analysis of genes dysregulated by over-expression, or knock-down of miR-146a in resting EOC 13.31 cells using either miRNA mimics or anti-miRs. **A.** Venn diagram to show the intersection between genes down-regulated by miR-146a over-expression, up-regulated on miR-146a knock-down and those targets bioinformatically predicted using the TargetScan 5.1 program and IPA software. **B.** Networks showing the interactions between several predicted miR-146a target genes. Shaded grey are those genes also bioinformatically predicted using the TargetScan 5.1 program and IPA software.

It has been previously reported that most translational repression is accompanied by miRNA-mediated mRNA degradation, we therefore used this assumption to predict potential direct targets of miR-146a in this system [34], [35]. We determined the intersection between miRNA targets bioinformatically predicted by Targetscan 5.1, mRNAs down-regulated following miR-146a over-expression, and mRNAs up-regulated following miR-146a knock-down. The results are summarized in Figure 5A and Table 3 provides a list of the genes that were common. A network of the deregulated genes that are also potential miR-146a direct targets is provided in Figure 5B. This includes the down-regulation of the cytokine IL-1B by miR-146a over-expression and up-regulation following knock-down. The gene IL-1B has previously been reported to be negatively regulated by miR-146a [30].

**Table 3:** Analysis of genes dysregulated by over-expression, or knock-down, in resting EOC 13.31 cells using either miR-146a mimics or anti-miRs.

Symbol	Entrez Gene Name
ATP2A2	ATPase, Ca++ transporting, cardiac muscle, slow twitch 2
CACNB2	calcium channel, voltage-dependent, beta 2 subunit
**CASK**	calcium/calmodulin-dependent serine protein kinase
CDC42ER2	CDC42 effector protein (Rho GTPase binding) 2
CDK7	cyclin-dependent kinase 7
CHRNA1	acetylcholine receptor subunit alpha-1-A
**CPSF2**	cleavage and polyadenylation specific factor 2
CYP2B6	E74-like factor 1 (ets domain transcription factor)
**DNAH1**	dynein, axonemal, heavy chain 1
EDEM2	ER degradation enhancer, mannosidase alpha-like 2
EPHA5	Eph receptor A5
GALM	galactose mutarotase (aldose 1-epimerase)
GATA6	GATA binding protein 6
**GFAP**	glial fibrillary acidic protein
GHSR	growth hormone secretagogue receptor
GLI3	GLI family zinc finger 3
**GRIA3**	glutamate receptor, ionotrophic, AMPA
**HIPK2**	homeodomain interacting protein kinase 2
**HLA-A**	major histocompatibility complex, class I, A
**HLA-B**	major histocompatibility complex, class I, C
**HLA-C**	major histocompatibility complex, class I, C
IL1B	interleukin-1-beta
IRF6	interferon regulatory factor 6
**KALRN**	kalirin, RhoGEF kinase
**KCNJ5**	potassium inwardly-rectifying channel, subfamily J, member 5
KDR	kinase insert domain receptor
KLK10	kallikrein-related peptidase
**MLLT4**	myeloid/lymphoid or mixed-lineage leukemia
NRG1	neuregulin 1
**NTRK2**	neurotrophic tyrosine kinase, receptor, type 2
**PARD6B**	par-6 (partitioning defective 6) homolog beta
**RRBP1**	ribosome binding protein 1 homolog
RYR3	ryanodine receptor 3
**SMAD3**	SMAD family member 3
TMSB4X	thymosin-like 2
TUBA1C	tubulin, alpha 1c
**TYRP1**	tyrosinase-related protein 1

Genes listed in bold are those that are bioinformatically predicted to be targets of miR-146a using the TargetScan 5.1 program and IPA software.

### Transcription of genes involved in NF-κB, JNK and MAPK signaling pathways and the expression of inflammatory mediators are modulated by miR-146a expression in TLR2 stimulated microglia

We hypothesized that the majority of miR-146a targets that act as important microglial immune response regulators would likely only be evident during conditions in which cells were stimulated by a ligand. To this end we transfected EOC 13.31 cells with miR-146a anti-miRs or mimics prior to stimulation of TLR2. Transfected anti-miRs or mimics were stably detected over a 24–48 hour period post transfection; therefore cells were transfected and allowed to recover overnight, thus reducing the likelihood of differential expression induced by the transfection process, prior to stimulation. We initially transfected a miR-146a anti-miR as a means to determine genes whose expression was potentially relieved, either directly or indirectly, by a reduction in miR-146a. RNA was extracted at 8 hours and 24 hours following LPS treatment and the transcriptional response was determined by microarray analysis in comparison with mock-treated controls. Indeed, we did find a significant number of up-regulated genes at 8 hours (691 annotated genes), with a smaller number (354 annotated genes) up-regulated at 24 hours post stimulation. As expected, a great deal of consistency was evident between the two data sets, 287 of the 354 genes up-regulated at 24 hours were also up-regulated at 8 hours. Very few genes exhibited significant down-regulation following this treatment; only 6 at 8 hours post stimulation, and 2 at 24 hours, both of these two genes were amongst the 6 genes identified at 8 hours confirming very high reproducibility of the microarray data. Examination of the ontologies enriched in this set of up-regulated genes showed genes known to play a role in infection processes, including NR3C1, PTPRC, SFRS1, SFRS5, SMAD3, C3 and DDX58. Other groups of up-regulated genes included genes involved in cell cycle progression, cell death, cellular movement and morphology. A number of important transcriptional regulators PAX6, MDM2, JAK2 and SMAS3 were increased, as were some caspases, CASP7 and CASP8, and the apoptotic regulator BCL2L1. Caspase-8 is known to have non-apoptotic functions and has been shown to be required for TLR signaling and in the regulation of NF-κB function [36].

**Figure 6:**
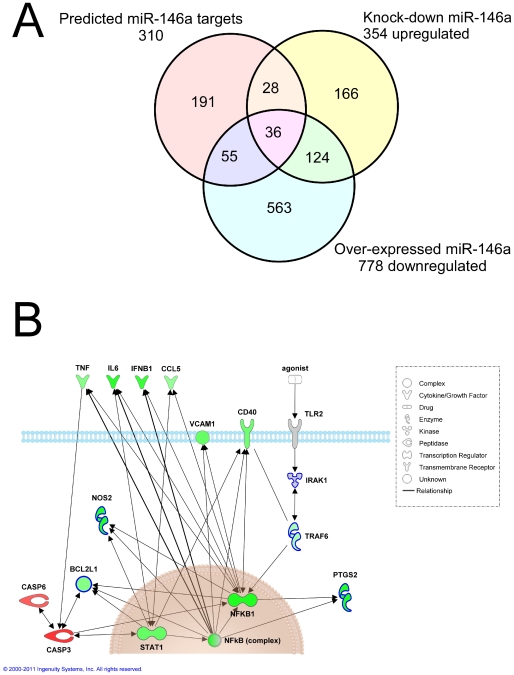
Analysis of genes dysregulated upon over-expression, or knock-down, of miR-146a in stimulated EOC 13.31 cells using either miRNA mimics or anti-miRs. **A.** Venn diagram to show the intersection between genes down-regulated in LPS stimulated EOC 13.31 cells by miR-146a over-expression, and those up-regulated upon miR-146a knock-down. Also shown are those targets bioinformatically predicted using the TargetScan 5.1 program and IPA software. **B.** Schematic showing alterations in expression in key inflammatory response-related genes in LPS stimulated EOC 13.31 cells following miR-146a over-expression. Colored green are those down-regulated genes, colored red are up-regulated genes, while those highlighted in blue are bioinformatically predicted targets of miR-146a using the TargetScan 5.1 program and IPA software.

Similarly, over-expression of miR-146a was achieved by transfection of a miRNA mimic and LPS stimulation was performed 24 hours later, followed by transcriptional analysis at 8 and 24 hours. A significant number of genes were de-regulated under these conditions, 22 (3 down-regulated, 19 up-regulated) at 8 hours post transfection and 977 (778 down-regulated, 199 up-regulated) at 24 hours. At the 8 hour time-point the cells over-expressing miR-146a share very similar transcriptional profiles with mock-transfected, LPS stimulated cells. However, at the later time-point of 24 hours post stimulation, significant changes in the expression of multiple genes are observed relative to the controls. A diagram depicting the expression data produced 24 hours post-stimulation is provided in Figure 6A. Analysis of the biological functions of those genes modulated by miR-146a expression revealed primarily the down-regulation of immune response effector genes in comparison to the mock-transfected, LPS stimulated cells. Signaling pathways involving NF-κB, JNK and MAPK are implicated in Figure 6B.

**Figure 7:**
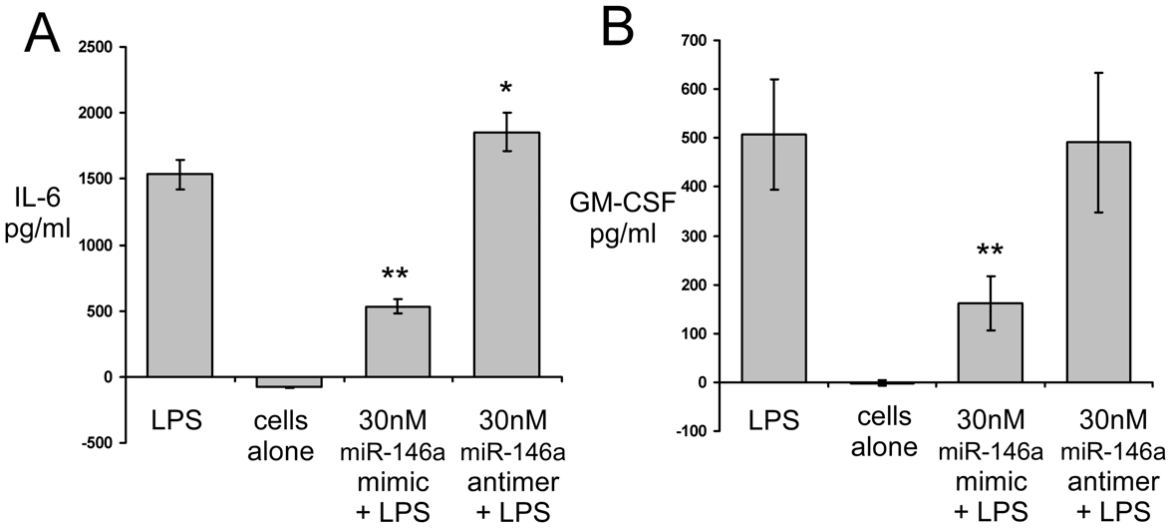
EOC 13.31 cells were transfected with 30 nM of miR-146a mimic or anti-miR and 16 hrs later stimulated with 10 ng/ml LPS. At 24 hours post LPS stimulation, culture supernatant was collected and the levels of IL-6 and GM-CSF (coded for by the gene CSF2) were determined by ELISA; significance: ** p<0.001, * p<0.01.

To confirm the down-regulation of immune effectors at the protein level we used ELISAs to determine IL-6 and GM-CSF (CSF2) levels upon TLR stimulation in miR-146 over-expressing cells. Production of these cytokines was significantly reduced by miR-146a over-expression upon TLR2 stimulation of EOC 13.31 cells versus cells transfected with a scrambled control RNA (Figure 7). Highly significant (p<0.001) down-regulation of IL-6 and GM-CSF following miR-146a over-expression correlated well with our genomic data. In addition, transfection of a miR-146a anti-miR resulted in a small but significant up-regulation of IL-6 in these cells, presumably correlating with a reduction of endogenous miR-146a by anti-miR sequestration in EOC 13.31 cells. One up-regulated cytokine coding gene was IL-10, a negative regulator of cytokine production during the immune response; however, this gene was expressed at very low levels based on the spot intensity observed by microarray and we were unable to detect IL-10 expression by ELISA. Table 4 provides a summary list of some of these inflammation-related genes that appear altered in response to increased expression of miR-146a, in the process illustrating a potential dampening effect of the transcriptional response to stimulation.

**Table 4:** Inflammation-related genes that are altered in response to increased expression of miR-146a in LPS stimulated EOC 13.31 cells.

***ADM***	***CD44***	***CXCL10***	***FOS***	***IFNA5***	***IRGM***	***MX2***	***SLC7A2***	***TRIM21***
AP3D1	***CD69***	***CXCL11***	FPR2	***IFNB1***	***ITGAM***	***MYC***	SLPI	***TRPC2***
***BCL2L1***	***CEBPB***	***CXCL3***	***GBP2***	***IKBKE***	KITLG	***NCF1***	***SMAD3***	***TSLP***
CASP3	***CEBPG***	***CXCL6***	***GIMAP5***	IL10	***LCN2***	***NFATC3***	***STAT1***	***VCAM1***
***CBLB***	***CHUK***	***CXCL9***	HP	IL12B	***LCP2***	***NFKB1***	***STAT2***	***ZCCHC11***
***CCL20***	CIITA	***DLG1***	***ICOSLG***	***IL15***	***LTA***	***NOS2***	TGFBR3	
***CCL3***	***CSF2***	***DOCK2***	***IER3***	***IL16***	***MALT1***	***PDCD1***	***TLR2***	
***CCL5***	***CSF3***	***DPP8***	***IFI44L***	***IL1A***	***MAP4K2***	***PF4***	***TLR3***	
***CD274***	***CSK***	***ERAP1***	***IFI47***	***IL1B***	MGST1	***PTGS2***	***TNF***	
***CD28***	***CX3CL1***	***EREG***	***IFIH1***	***IL1RN***	***MSH2***	RNASEL	***TNFSF10***	
***CD40***	***CXCL1***	***FAS***	***IFNA16***	***IL6***	***MX1***	***S100A10***	TP53	

Down-regulated genes are in bold italics, up-regulated genes are in plain type.

Direct targets of miR-146a important in immune activation are likely to be those found at the intersection of genes that are up-regulated following knock-down and down-regulated after over-expression, provided that they exhibit altered expression that is detectable at the mRNA level. We found that 36 genes, out of a total of 160 significantly de-regulated genes whose expression followed this pattern, that were also bioinformatically predicted targets of miR-146a using the TargetScan 5.1 program and IPA software. These are listed in Table 5. The transcription regulator SMAD3 was identified as a potential target of miR-146a in both resting cells and those stimulated with LPS and has not been described to be a target of miR-146a. In addition, NOS2, QK1 and BCL2L1 are also predicted from this study as being strong candidates for targeting by miR-146a that have not previously been described. They are also highly related based on potential interactions identified using the IPA tool, potentially a co-ordinately regulated gene set. Previously, TRAF6, IRAK1 and IRAK2 have been identified and validated as putative targets of miR-146a in macrophages [16], [37]. These targets have been shown to be regulated primarily at the protein level, and in accordance they were not identified during this transcription based analysis; however, a small but significant decrease in TRAF6 gene expression in miR-146a transfected and LPS stimulated cells versus control was indeed observed in our work.

**Table 5:** Analysis of genes dysregulated by over-expression, or knock-down, of miR-146a in LPS stimulated EOC 13.31 cells using either miRNA mimics or anti-miRs.

Symbol	Entrez Gene Name
AMPD3	adenosine monophosphate deaminase (isoform E)
ARCN1	archain 1
BCL2L1	BCL2-like 1
CSDE1	cold shock domain containing E1, RNA-binding
CSTF2	cleavage stimulation factor, 3′ pre-RNA, subunit 2, 64 kDa
DARS	aspartyl-tRNA synthetase
DHRS9	dehydrogenase/reductase (SDR family) member 9
ELF1	E74-like factor 1 (ets domain transcription factor)
FAM107B	family with sequence similarity 107, member B
FUBP1	far upstream element (FUSE) binding protein 1
HMGCS1	3-hydroxy-3-methylglutaryl-Coenzyme A synthase 1
IFIT2	interferon-induced protein with tetratricopeptide repeats 2
IRS1	insulin receptor substrate 1
MED1	mediator complex subunit 1
NOS2	nitric oxide synthase 2, inducible
PCTK2	PCTAIRE protein kinase 2
POGK	pogo transposable element with KRAB domain
PSME4	proteasome (prosome, macropain) activator subunit 4
QKI	quaking homolog, KH domain RNA binding
QSER1	glutamine and serine rich 1
RAB8B	RAB8B, member RAS oncogene family
RAI14	retinoic acid induced 14
SEC23A	Sec23 homolog A
SH3BGRL	SH3 domain binding glutamic acid-rich protein like
SLC2A3	solute carrier family 2 (facilitated glucose transporter), member 3
SMAD3	SMAD family member 3
SOX10	SRY (sex determining region Y)-box 10
TAF9B	TAF9B RNA polymerase II, TATA box binding protein (TBP)-associated factor, 31 kDa
TFCP2L1	transcription factor CP2-like 1
UBE2K	ubiquitin-conjugating enzyme E2K (UBC1 homolog, yeast)

Genes listed are those that are bioinformatically predicted to be targets of miR-146a using the TargetScan 5.1 program and IPA software.

## Discussion

Microglial cells, the brain's resident immune effector cells, respond to prion deposition by the conversion of a resting phenotype into one that is ‘activated’. However, although they take on an activated phenotype they synthesize fairly low levels of pro-inflammatory cytokines; presumably as a defense mechanism to prevent the severe pathology that can arise in host tissue as a result of an acute inflammatory response induced by rampant signaling in phagocytes. The specific stimuli and signaling pathways that lead to this modulation of functions are as yet unknown; however, it is evident that tight regulation exists in activated microglia modulating their production of pro-inflammatory cytokines although phenotypic changes are evident that are indicative of phagocytic capability. It is yet to be determined whether microglial activation reflects a direct response to prion toxicity or a secondary response to clear cell debris following neuronal damage and death. However, it is clear that phenotypic changes in microglia are one of the earliest apparent alterations in tissue pathology detectable during disease.

Firstly, we confirmed that miR-146a is over-expressed in the brain's of mice during scrapie infection and that this expression is, most likely, highly enriched in cells of microglial origin based on miRNA expression profiling of various brain-derived cell lines. Nevertheless, it is also possible miR-146a is expressed at low levels in other cell types. In subsequent studies using TaqMan® qRT-PCR assays we have found that miR-146a is expressed at low levels in laser capture microdissected (LCM) neurons isolated from mouse hippocampus (data not shown) and the expression and/or induction of this miRNA in neurons is therefore a strong possibility. Additionally, a number of miRNAs found to be up-regulated in a recent study profiling glial cells in multiple sclerosis brain lesions, as well as our previous study to determine deregulated miRNAs in scrapie infection, were also identified to be enriched in microglial cell lines in this study [18], [38]. These were miR-142-3p, miR-34a, and miR-223. Further work to determine whether these miRNAs also play a role in the regulation of innate immunity, NOS production or phagocytosis was beyond the scope of the present study.

Initially, we attempted to use oligomeric recombinant PrP or the neurotoxic peptide, PrP^106–126^, to induce activation of miR-146a induction in several microglial cell lines, however, this proved to be unsuccessful, as we observed little to no activation of this miRNA (data not shown). The limited transcriptional response to prions in cultured microglia, and to a certain extent cultured neuronal cells, is in stark contrast with the robust transcriptional responses that is often obtained from whole brain tissue. This discrepancy is very likely multi-factorial and contributing factors include, but are certainly not limited to, lack of multiple cell types and associated cell-to-cell signaling in *in vitro* culture, lack of normal cellular functions under *in vitro* conditions, and also the time required for disease progression, which is often months to years in whole organism/animal. It is often speculated that the *in vivo* transcriptional response likely represents the host's response to the process of neurodegeneration as much, if not more than, the response specifically to prions, which may account for our inability to detect a robust miR-146a induction in the cultured microglia. Due to this limitation, we therefore used microarray analysis to determine which microglial surface receptors are over-expressed in the brains of an RML scrapie model of prion disease and therefore likely to play a pertinent role in the activation state of microglia.

TLR2 appeared to be the most up-regulated and abundant receptor in our model although other receptors including Fc (FCGR1 and FCGR2) and complement receptors (ITGAM and ITGAX) were also found to be over-expressed. Further investigation into whether stimulation of these receptors is involved in prion pathobiology is warranted. Contrary to previous reports, we did not find the scavenger receptors SCARB1, SCARB2 and AGER to be up-regulated during disease [39]. Interestingly TLR2, and TLR7 which was also up-regulated in our prion model, has also been shown to display profoundly increased expression levels in APP transgenic mice that accumulate amyloid deposits in their brain [40]. Furthermore, an injection of amyloid into the hippocampus provokes increased TLR2 expression [41]. It has previously been reported that TLR4 is also increased in prion models, although in our study the transcript coding for TLR4 was below the level of detection. Further evidence that TLRs may be important in the regulation of the microglial response in prion diseases stems from the observation that mice with defective TLR4 signaling, making them hyporesponsive to LPS, exhibit an accelerated rate of prion disease [11]. This implies a protective role for TLR stimulation of microglia during disease. In Alzheimer's disease, a pathology in which an oligomeric form of beta amyloid (Aβ) builds up in a similar fashion to PrP^Sc^ in prion disease, phagocytosis of Aβ by activated microglia is significantly increased in the presence of ligands that activate TLRs [42]–[44]. Landreth and Reed-Geaghan (2009) have also shown that the response of microglia to fibrillar Aβ is reliant upon the expression of TLR4, TLR2 and the co-receptor CD14 to activate intracellular signaling [45]. Cells lacking these receptors could not initiate a Src-Vav-Rac signaling cascade required to stimulate phagocytosis of amyloid beta deposits [45].

TLR4 stimulation of the BV-2 microglial cell-line resulted in activation and in the transcription of miR-146a, as did stimulation with the TLR2 agonist HKLM. EOC 13.31 cells that lack a functional TLR4 receptor could also be induced to over-express miR-146a on stimulation with a TLR2-agonist. Our first finding was that in contrast to the rapid and transient induction of transcription of inflammatory mediators such as cytokines IL-6 and TNF, miR-146a expression follows alternate kinetics. MiR-146a was induced at a slower rate, reaching a peak after 24 hours that is maintained even at 48 and 72 hours post-stimulation; time-points when cytokine expression levels have returned to base-line. We hypothesize that miR-146a may be involved in a prolonged dampening of the innate immune response following activation. MiR-146a may also contribute to the establishment of a microglial state primed in some way for response to repeated exposure to immune activators, as has recently been suggested to occur in macrophages [31].

To more fully understand the transcriptional response modulated by miR-146a, we performed knock-down or over-expression experiments on cells either in resting or TLR-stimulated state. We sought to determine the full extent of changes elicited by miR-146a, not just direct targets but also those transcripts that are down-stream of the direct targets. Whole pathways may be down-regulated, for example, if a receptor is a direct target. Alternatively, up-regulated mRNAs may perhaps indicate that the miRNA target is a negative regulator of transcription or of a signaling pathway. Knock-down may produce the opposite effect in some instances if basal levels of miRNAs are exerting a regulatory effect.

A functional genomic analysis using over-expression and knock-down of miR-146a in ‘resting’ EOC 13.31 cells or following stimulation of TLR revealed significant alteration of numerous transcripts. Notably, these included numerous genes important to phagocyte function including genes involved in the inflammatory response (the production of cytokines and prostaglandins) as well as genes involved in phagocytosis including the cellular morphological changes that accompany activation and the oxidative burst. It was interesting to observe the down-regulation of important phagocytosis related genes such as IL-1B, iNOS and CYBA (part of the NADPH oxidase complex which is the primary source of reactive oxygen intermediates produced during oxidative burst) in resting cells. In particular, IL-1B, a key mediator of the inflammatory response, is both down-regulated following miR-146a over-expression and up-regulated after knock-down. This suggests that the basal level expression of IL-1B in microglia is modulated, in part, by miR-146a.

Other significant alterations in expression include downstream mediators of the pro-inflammatory transcription factor, nuclear factor-kappa B (NF-κB) and the JAK-STAT signaling pathway (Figure 4B). Previous studies have determined that miR-146a can regulate NF-κB itself, as well as key genes downstream of NF-κB [16]. MiR-146a is also transcribed by NF-κB and therefore may operate as part of a feedback inhibitory regulatory-loop to modulate inflammation by this transcription factor [46]. Validated miR-146a targets include two genes that are important mediators of TLR signaling [16]. These genes are IRAK1, which is in part responsible for transcription of NF-κB in response to immune stimulation, and TRAF6. The 3′UTRs of these genes are responsive to interaction with miR-146a, however, regulation was shown to be at the level of translation; mRNA levels remained unchanged. The mRNA levels of IRAK1 were similarly found to be unchanged in our study, although we measured a small, but significant, down-regulation of TRAF6 gene expression in EOC 13.31 cells transfected with a miR-146a mimic.

Expression of the complement factor H (CFH) gene was another of those genes down-regulated on application of miR-146a mimic. Interestingly, this gene has been shown to be targeted by miR-146a and down-regulated in Alzheimer's disease as well as in other diseases such as Herpes simplex virus type1 infection [47], [48]. It was also intriguing to note that amongst the up-regulated genes was a highly significant group of ontologically related genes involved in small RNA processing. These included TNRC6A and TNRC6B, two important proteins found in P-bodies associated with Argonaute [49]. This finding could potentially reflect a function of miR-146a in the regulation of P-body function and processing of miRNAs themselves, perhaps by relieving miR-146a inhibition of a transcriptional repressor. An alternative explanation is that this may reflect a non-specific cellular response to the transfection of miRNA in the cell.

We hypothesized that miR-146a targets, that directly modulate an immune response, would likely only be apparent following an immune challenge. This was indeed the case as many more de-regulated genes were evident upon the over-expression or knock-down of miR-146a following TLR stimulation. Most notable was the expanded list of cytokines and inflammatory mediators whose transcripts were significantly decreased in comparison to the levels measured in mock-transfected, stimulated, microglial cells. The primary effector mechanism of innate-immunity is the generation of acute and chronic inflammatory responses notably through regulation of cytokines IL-1B and IL-18. Interestingly, IL-1B appeared to be tightly regulated by miR-146a; its expression is decreased following transfection of the miR-146a mimic and increased following knock-down with an anti-miR in both resting and TLR-stimulated cells. Knock-down also appeared to relieve expression of the IL-18 receptor; IL-18 being another key mediator of the inflammatory response to tissue damage.

Conversely, we found a small number of genes to increase in expression upon transfection of a miR-146a mimic. One of these was IL-10, an anti-inflammatory cytokine that can down-regulate the expression of cytokines, MHC class II molecules, and co-stimulatory molecules on macrophages, and has been found to be up-regulated in human patients with CJD [50]. Additionally, it can block NF-κB activity, and is involved in the regulation of the JAK-STAT signaling pathway. The ability of IL-10 to decrease the production of pro-inflammatory cytokines has been suggested as a potential protective role in neurodegenerative diseases. Other anti-inflammatory cytokines exist, such as IL-4, IL-13 or TGF-β, however, no relative change in the expression levels of these cytokines was observed in LPS treated microglia following over-expression of miR-146a. However, a TGF-β activated transcription factor SMAD3 was identified as a potential miR-146a target in both resting and LPS stimulated cells, suggesting a possible role for SMAD3 in microglial activation. SMAD3 knock-out mice exhibit alterations in many aspects of glial function, including altered scar formation and immune response to injury [51]. Fewer neutrophils, macrophages/microglia, NG2-positive cells and GFAP-positive cells were reported around lesions in Smad3 null mice brains. Therefore, down-regulation of SMAD3 by miR-146a expression could well be an important mechanism to modulate immune response and glial migration.

Given that TLR2 is extremely abundant and highly up-regulated upon prion infection, we hypothesize that signaling through this TLR plays a role during disease. MyD88 is the main adaptor protein implicated in TLR signaling and is considered essential for the induction of inflammatory cytokines triggered by most TLRs, including TLR2. Nevertheless, the study determined by Prinz *et al*. (2003) showed that MyD88 knock-out mice were readily infectable with scrapie and did not show any significant alteration in the time-course or pathology of the disease [12]. This data implies that TLR2-signaling is not essential to the disease process and that our model may not mimic processes that occur during disease. However, effector molecules downstream of multiple cell surface receptors, including TLR2 (and TLR4) signals, are similar and the action of miR-146a to modulate the innate immune response and microglial activation in these model systems is likely to be indicative, despite the original upstream signal. In addition, recent studies have shown that TLR2 stimulation is indeed capable of eliciting a MyD88- and TRIF-independent inflammatory cytokine response [52]. Some evidence also exists suggesting that this response is independent of phagocytic clearance pathways that appear to be MyD88 dependent [53]. Interestingly, in a mouse model of Alzheimer's disease, MyD88 deficient and APP over-expressing mice had an accelerated disease process pointing to a role for MyD88 in clearance of amyloid [54]. It has also been reported by others that TLR 2, 4, and 9 signaling modulates phacogytosis and clearance of neurotoxic amyloid deposition in neurodegenerative diseases, perhaps by MyD88 independent signaling pathways [43], [55]. Those genes that are involved in phagocytosis and morphological changes that are altered in response to miR-146a over-expression may therefore be important to these processes. Studies also link a reduction in TLR2 expression with an increase in phagocytosis, implying an inverse relation between TLR2 expression and phagocytic activity. In terms of prion disease this suggests the considerable increase in expression of TLR2 observed at clinical stages of prion disease could be indicative of the accumulation of microglia that are not in an activation state that is optimal for phagocytosis. This could therefore contribute to defective clearing of debris and prion accumulation during later stages of disease. Investigation into whether TLR2 signaling is indeed important in prion disease pathology, and the identification of signaling pathways important in microglial activation independent of MyD88, warrants further investigation.

**Figure 8:**
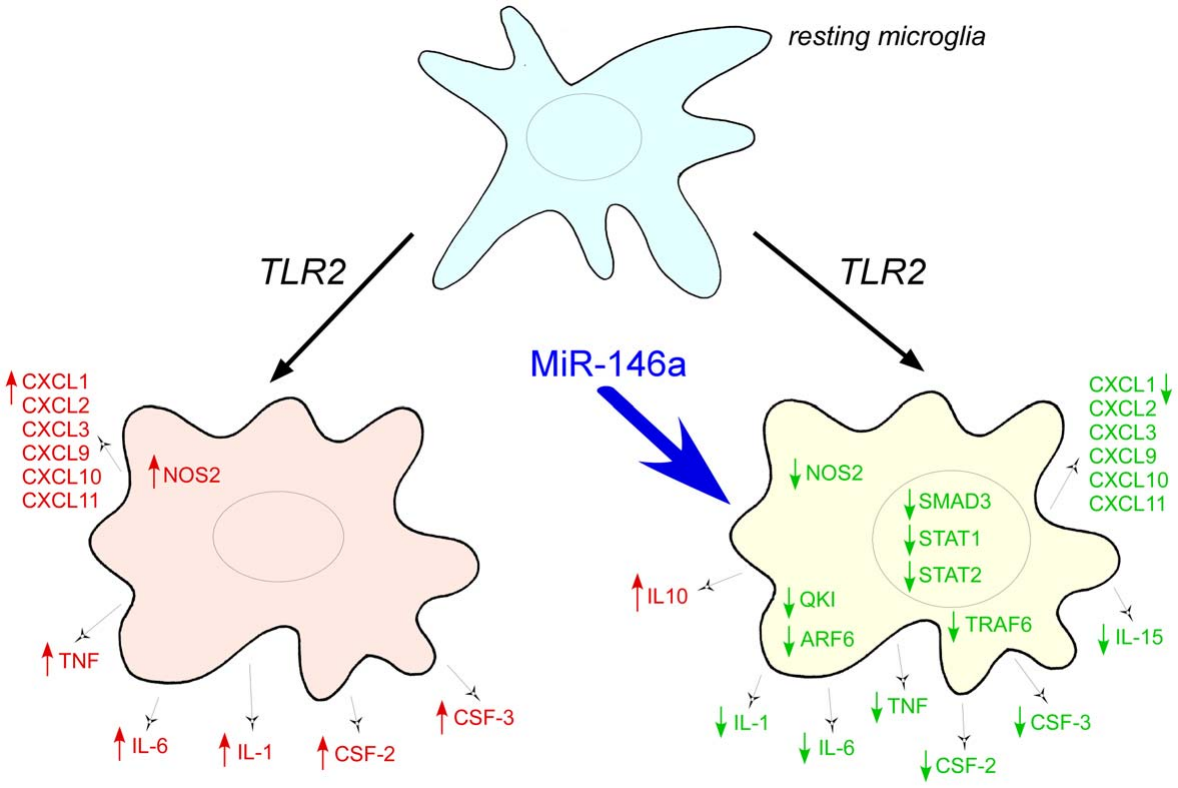
A summary showing key inflammatory response-related genes whose expression is modulated upon the over-expression of miR-146a.

Ultimately, therapeutic modulation of the immune system in the brain, perhaps by anti-miRNA strategies to repress the effects of up-regulated miRNAs, is a goal for treatment of progressive neurodegeneration. At a simplistic level knocking-down microRNAs such as miR-146a, whose increased expression correlates with disease progression, may present a treatment strategy. On the other hand, this up-regulation may, in fact, be providing protection from potentially harmful immune stimulation and knock-down may be counter productive. We initially hypothesized that given the immunomodulatory role ascribed to miR-146a in macrophages, the up-regulation of miR-146a in scrapie infected brain may reflect a role in keeping the pro-inflammatory response of microglia to prion replication and degeneration “in-check” and be beneficial to the course of disease. We show here that miR-146a appears to have an overarching role in microglial function Figure 8. Not only are genes involved in cytokine and other aspects of the inflammatory response regulated by its expression, but we found in this study that genes involved in morphological changes that accompany activation, chemotaxis and oxidative burst-related genes may also be targets for either direct or indirect miR-146a regulation. Therefore, altering miR-146a levels in a prolonged therapeutic attack against neurodegenerative processes may have unforeseen consequences such as altering the phagocytic potential of microglia to remove toxic protein inclusions. In summary antisense-miRNA therapeutic strategies offer huge promise over siRNA strategies because of the potential to affect the regulation of multiple disease-related genes simultaneously. However, studies such as this, to define the exact sphere of influence of a miRNA targeting multiple gene networks are essential to assessing their ultimate therapeutic potential.
